# Preparedness for paediatric cardiopulmonary resuscitation amongst medical doctors working in primary health care facilities in Cape Town, South Africa

**DOI:** 10.4102/safp.v64i1.5323

**Published:** 2022-01-26

**Authors:** Nabeela Amien, Graham Bresick, Katya Evans

**Affiliations:** 1 Department of Family Medicine, Faculty of Health Sciences, University of Cape Town, Cape Town, South Africa; 2 Division of Emergency Medicine, Faculty of Health Sciences, University of Cape Town, Cape Town, South Africa

**Keywords:** paediatric, CPR, knowledge, confidence, equipment availability

## Abstract

**Background:**

Cardiopulmonary resuscitation (CPR) is the principal medical intervention used to reduce the high mortality associated with the cardiorespiratory arrest. There is a paucity of literature on the preparedness for paediatric cardiopulmonary resuscitation (pCPR) amongst doctors in Cape Town. This study aimed to assess the preparedness for pCPR of doctors working in Western Cape Provincial Government primary health care facilities (PHCFs) in Cape Town with regard to knowledge, confidence and doctors’ knowledge of equipment availability.

**Methods:**

A cross-sectional study using a questionnaire to collect quantitative data from a sample of 206 doctors working in Cape Town PHCFs.

**Results:**

The questionnaire was completed by 173 doctors (84% response rate). The majority (81.8%) had not undergone pCPR training (Paediatric Advanced Life Support or Advanced Paediatric Life Support). Basic life support was done by 88.3%: 28% greater than two years ago. The average pCPR knowledge score was 61% (standard deviation [s.d.]: 20.3, range: 8.3% – 100%). Doctors in their community service and internship years had significantly higher knowledge scores compared to grade 3 Medical officers (*p* = 0.001 and *p* = 0.010, respectively). Eleven per cent had performed pCPR > 10 times in the past year; 20% had never performed pCPR and 35% did not feel confident performing pCPR. More than 35% of doctors were uncertain about the availability of equipment in their facility.

**Conclusion:**

Doctors working in Cape Town PHCFs have poor knowledge, have low confidence levels and are poorly prepared to perform pCPR. Urgent attention needs to be given to ensuring formal pCPR training and acquaintance with equipment availability and location in Cape Town PHCFs.

## Introduction

Although paediatric cardiorespiratory arrest is a rare event,^1^ it is associated with high mortality. In cardiorespiratory arrest, cardiopulmonary resuscitation (CPR) can be an important life-saving measure because when performed correctly, patient outcomes are better.^2^ Given that paediatric cardiorespiratory arrest has a high mortality, doctors must be equipped to perform paediatric cardiopulmonary resuscitation (pCPR) correctly and timeously using a structured evidence-based approach. Three critical aspects are associated with performing effective pCPR, namely doctors’ knowledge of how to perform pCPR, their confidence levels and their knowledge of the availability of the required equipment in their facility.^2,3,4^

Cardiopulmonary resuscitation skills have a positive impact on the return of spontaneous circulation and neurological outcomes, and this knowledge should be shared in the form of teaching and ongoing simulated practices.^4^ There is a proven link between knowledge of the CPR guidelines and the likelihood of being able to follow and perform the guidelines correctly.^4,5,6^ Multiple studies demonstrate that doctors working in clinical settings where pCPR may be required lack the knowledge levels required to perform it effectively.^7,8^ Furthermore, medical professionals are only able to retain the knowledge of CPR guidelines for short amount of time. This occurs as early as 3–6 months post-training^8^ and ultimately results in a decrease in the quality of CPR^9^ and poorer patient outcomes.^10^

Confidence levels amongst doctors concerning pCPR can be defined as their belief in their ability to perform effective CPR on paediatric patients.^11^ Positive correlations have been found between confidence levels in performing pCPR and attending a pCPR course, as well as with seniority level of doctor.^11^ No literature has explored confidence in performing pCPR in different aspects such as anxiety levels, specific CPR courses and experience levels across broader groups of doctors. Therefore, a more in-depth understanding of how self-confidence impacts preparedness for pCPR is necessary.

Equipment availability is an important precursor in being able to perform pCPR adequately. All doctors should be familiar with the equipment as well as its location.^12^ No studies were found that examined doctors’ knowledge regarding equipment availability. Such knowledge is integral to the chain of survival.

There is currently a paucity of data on the incidence and outcomes of pCPR in Cape Town. South Africa has a high level of income inequality^13^ and high trauma rates^13^; both have been linked to poor health outcomes including higher infant mortality rates.^14^ It can, therefore, be postulated that South African doctors may see more cases of paediatric cardiorespiratory arrest. Thus, it is imperative that South African doctors working in the public healthcare system be adequately prepared to perform pCPR.

The aims and objectives of the study were to assess the preparedness for pCPR of medical doctors working in primary health care facilities (PHCFs) in the Cape Metropole of the Western Cape (WC). Preparedness was defined as sufficient knowledge and confidence to perform pCPR competently and knowledge of the availability of the necessary equipment.

## Research methods and design

### Study design

This is a cross-sectional study using a questionnaire.

### Setting

The study was conducted at community health centres (CHCs) and community day centres (CDCs) situated within the four district health substructures in the Cape Town Metropole. These are PHCFs without any overnight beds. Community health centres have 24-h emergency centres, whereas CDCs have emergency centres open from 8:00 to 16:00. The facilities included in the study with the number of doctors employed (in brackets) are as follows:

- Community day centres: Bellville (2), Bishop Lavis (5), Bothasig (2), Greenpoint (3), Ravensmead (2), Ruyterwacht (2), District 6 (6), Kensington (2), Maitland (1), Crossroads (5), Dr Abduragman (4), Heideveld (5), Nyanga (1), Mfuleni (5), Nomzamo (4).
- Community health centres: Delft (13), Elsies River (6), Goodwood (2), Kraaifontein (10), Retreat (13), Vanguard (12), Hanover Park (9), Inzame Zabantu (1), Mitchells Plain (11), Khayelitsha (12), Michael Mapongwana (11), Nolungile (5).

### Study population and sampling strategy

The study population included 173 of 206 doctors working in CHCs and CDCs in the Cape Metropole. The doctors who participated comprised interns (general practitioners in training who have completed medical school and have a medical degree but do not yet have a license to practise medicine unsupervised),^15^ community service doctors (general practitioners who have completed internship but still need to work one or two years in a state-run facility before being allowed to practise as a doctor independently)^16^ and medical officer (MO) grades 1–3 (general practitioners who have registered as independent practitioners grade 1 MOs have been working for the state for less than five years, a grade 2 MO for a minimum of five years and a grade 3 MO for a minimum of ten years),^17^ family medicine registrars (an MO undergoing training to become a family physician)^17^ and family medicine consultants (family medicine registrars who have completed a training program and masters in family medicine at a registered university in South Africa).^17^

The calculated sample size required to be statistically significant was 134 doctors, with a confidence interval of 95% and a margin of error of 5%. Each facility was attended until a total of 173 doctors were sampled. Each facility was sampled consecutively. Once approval had been received to conduct research per facility, the clinical managers of the respective facilities were contacted, and a time was arranged for the researcher to travel to the site and collect the data. Oversampling occurred as all doctors were present at facilities towards the end of data capturing.

Inclusion criteria:

- Registration with the HPCSA as a medical intern, community service MO, MO, family medicine registrar and a family medicine specialist. Doctors in these categories employed temporarily as locums were also included.
- Currently employed to provide clinical service at a CHC or CDC at the time of the survey completion.

Exclusion criteria:

- The City of Cape Town primary health care (PHC) facilities have been excluded from the study as these do not form part of the Cape Metropole.

### Data collection

The questionnaire was developed by the researcher as no suitable instrument measuring the three main variables in this study was found in the literature. The questionnaire comprised two sections:

- Section A focussed on demographics and background, specifically, what courses were completed by the participant, what their level of qualification is, how many times they have performed pCPR in the past year, how confident they are in their ability to perform pCPR and anxiety levels related to the performance of pCPR.
- Section B comprised knowledge-based questions related to pCPR as well as knowledge of equipment availability within the participants’ respective facilities. Key knowledge-based questions focussed on knowledge related to the management of the initial phase of pCPR (the pulse check, rate and ratio of rescue breathing and chest compressions as well as depth of chest compressions), knowledge related to defibrillation and adrenalin administration and management of ventricular fibrillation (VF), including refractory VF.

The knowledge questions were derived from the resuscitation of Southern Africa CPR algorithms.^18^ The questionnaire was piloted with five doctors (two emergency medicine physicians, two MOs and one community service doctor) working in a state-funded emergency room at Mitchells Plain District Hospital. Validity and reliability testing of the research tool were not done. This was because data collection on human participants was halted because of the COVID-19 pandemic just as initial data collection was completed.

During data collection, the researcher verbally requested the participation of the on-site doctors. Participants were introduced to the study both verbally and by means of an information sheet and subsequently signed a consent form. The doctors were then given a tablet/phone with the questionnaire on it, which was completed under the supervision of the researcher. All results were uploaded to the LimeSurvey site (https://limesurvey.org), a statistical survey app, in real-time and were transferred to Microsoft Excel for analysis.

### Data analysis

A biostatistician was consulted to analyse the results. Eleven results were removed because of incompletely filled questionnaires. One hundred and sixty two completed surveys were analysed. For assessing knowledge of pCPR amongst doctors, each question was coded as right or wrong and then a percentage knowledge score was obtained. Furthermore, to assess overall knowledge scores, a one-way analysis of variance (ANOVA) table was used to compare more than two groups. A Bonferroni corrected *p*-value was used to strictly adjust the level of significance so that the researcher did not interpret a difference when there was none. The same statistical analyses methods were used in assessing knowledge scores by doctors’ confidence levels. Independent sample *t*-tests compared knowledge scores of doctors who had or had not done each course.

## Results

### Characteristics of respondents

A total of 173 of 206 doctors working in PHCFs in the Cape Metropole participated in the survey, with a response rate of 83%. Twenty five per cent of them were MOs grade 1, and 30% of them were MO’s grades 2 and 3. Community service doctors made up 15% of the sample and interns made up 14%. Consultants and registrars each made up 8% of the sample (see Table 1). In terms of distribution of participants by subdistricts, 26% were from the Northern/Tygerberg subdistrict, 22% were from the Western/Southern subdistrict, 25% were from the Khayelitsha/Eastern subdistrict and 27% were from the Klipfontein/Mitchells Plain district.

**TABLE 1:** Grade of doctor.

Grade of doctor	*N*	%
Intern	22	13.6
Community service doctor	24	14.8
MO 1	40	24.7
MO 2	31	19.1
MO 3	18	11.1
Registrar	13	8.0
Consultant	14	8.6

**Total**	**162**	**100.0**

### Courses completed

**FIGURE 1:**
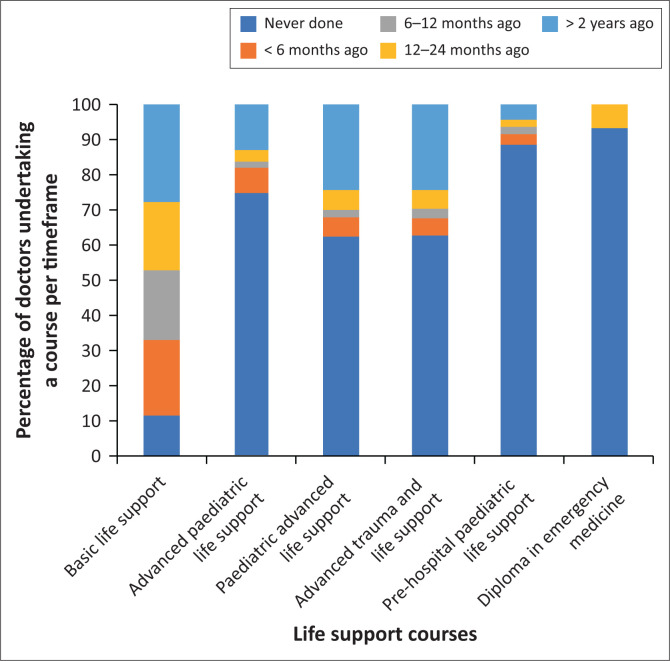
Distribution of life support courses done/not done by type of course.

Most doctors in the survey (almost 90%) had been on a basic life support (BLS) course, 28% completed it more than two (2) years prior to this survey (see Figure 1). Fifty three per cent of doctors said that the cost of attending a CPR course was a barrier.

A minority of doctors (25.3%) had done a course on advanced paediatric life support (APLS) and pre-hospital paediatric life support (PHPLS) (11.1%), as well as a diploma in emergency medicine (DIPEC) (6.2%). Moreover, only 37.0% of doctors had done a course on paediatric advanced life support (PALS) and advanced trauma and life support (ATLS). More consultants had attended a pCPR course (APLS 57.1%, PALS 64.3%) than any other grade of a doctor.

### Performance of cardiopulmonary resuscitation in the past year

In the past 12 months, 58% of doctors had performed pCPR 1–5 times, 12% (19) performed pCPR 6–10 times, 11% (17) had performed pCPR more than 10 times and 20% (31) of doctors had not performed pCPR.

### Knowledge of paediatric cardiopulmonary resuscitation

The average knowledge score was 61.7% (standard deviation [s.d.]: 20.3, range: 8.3% – 100%).

**FIGURE 2:**
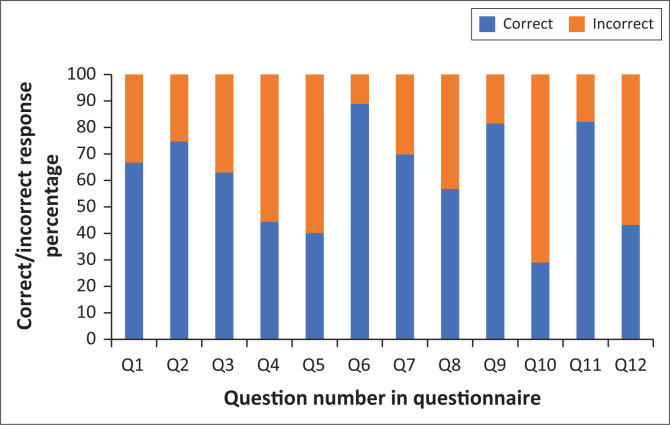
Knowledge scores of paediatric cardiopulmonary resuscitation per question.

Doctors were most likely to know the answer to knowledge questions 6, 9 and 11, and least likely to know the answers to questions 4, 5, 10 and 12 (see Figure 2 and Table 2). The average knowledge score was 61.7% (s.d.: 20.3, range: 8.3% – 100%).

**TABLE 2:** Knowledge questions.

Question number	Question asked
Q1	Where do you check for the pulse in a 1-year-old child?
Q2	Where do you check for the pulse in a 12-year-old child?
Q3	In an unresponsive paediatric patient, how long do you check the pulse for?
Q4	An unresponsive child presents to the emergency centre. The child is not breathing but has a pulse. At what rate do you commence rescue breathing?
Q5	An unresponsive 5-year-old child presents to the emergency centre. The child is not breathing but has a pulse of 120 beats per minute. You commence rescue breathing. At what heart rate would you need to initiate chest compressions on the child?
Q6	With regard to the child in question 11, what depth should the chest be compressed to ensure that your compressions are adequate?
Q7	When do you do the rhythm check during paediatric CPR?
Q8	What is the correct dosage of adrenalin for a paediatric cardiac arrest patient?
Q9	What is the ratio of compression to breaths in a non-intubated paediatric patient with two rescuers present?
Q10	What is the defibrillator dose for the second shock when defibrillating a paediatric patient? There is only one correct answer.
Q11	A child presents in cardiac arrest. Chest compressions are started and the defibrillator arrives. You check the rhythm and it looks like ventricular fibrillation. You shock the child. What do you do next? There is only one correct answer.

### Knowledge and category of doctor

A one-way ANOVA (see Table 3) revealed that knowledge scores differed significantly by the doctor category (*F* = 3.83, *p* = 0.001). Post-hoc comparisons revealed that community service doctors had significantly higher knowledge compared to MOs grade 3 (*p* = 0.001), as did interns (*p* = 0.010). Community service doctors had the highest knowledge scores, followed by interns, then consultants and registrars. Medical officers grade 1 and MOs grade 3 had the lowest knowledge scores.

**TABLE 3:** Knowledge scores by level of doctor category.

Level of doctor	*N*	Mean score (%)	s.d.	95% CI for mean		Min	Max
				Lower bound	Upper bound		
Community service doctor	24	71.9	17.2	64.6	79.1	33.3	100.0
Consultant	14	59.5	20.4	47.8	71.3	16.7	83.3
Intern	22	68.6	17.0	61.0	76.1	25.0	100.0
MO grade1	40	63.8	16.2	58.6	68.9	25.0	100.0
MO grade 2	31	57.3	22.0	49.2	65.3	8.3	91.7
MO grade 3	18	46.8	23.6	35.0	58.5	8.3	83.3
Registrar	13	58.3	20.4	46.0	70.7	25.0	100.0

**Total**	**162**	**61.7**	**20.3**	**58.5**	**64.8**	**8.3**	**100.0**

MO, medical officer; s.d., standard deviation; CI, confidence interval; Min, minimum; Max, maximum.

### Knowledge and course attendance

Knowledge scores did not differ depending on whether a doctor had previously done a course or not (all *p*-values > 0.05).

### Knowledge and confidence levels

Confidence levels were treated as a continuous entity during analysis. A one-way ANOVA (see Table 4) revealed that knowledge scores differed significantly by the doctor’s level of confidence (*F* = 5.12, *p* = 0.007). Post-hoc comparisons revealed that doctors who were confident had significantly higher knowledge compared to doctors who were not confident (*p* = 0.003), and doctors who were moderately confident had significantly higher knowledge compared to doctors who were not confident (*p* = 0.015). As can be seen from Table 4, knowledge scores decreased as confidence decreased.

**TABLE 4:** Knowledge scores by doctor’s confidence level.

Confidence level	*N*	Mean score (%)	s.d.	95% CI for mean		Min	Max
				Lower bound	Upper bound		
Confident	42	67.1	16.0	62.1	72.1	33.3	100.0
Moderately confident	64	63.9	20.5	58.8	69.0	8.3	100.0
Not confident	56	55.1	21.4	49.3	60.8	8.3	100.0

**Total**	**162**	**61.7**	**20.3**	**58.5**	**64.8**	**8.3**	**100.0**

MO, medical officer; s.d., standard deviation; CI, confidence interval; Min, minimum; Max, maximum.

### Knowledge and anxiety levels

Knowledge scores did not differ by doctors’ level of anxiety (*F* = 1.07, *p* = 0.344). Doctors with moderate anxiety appear to have higher knowledge than doctors with no or high anxiety, but this was also not significant (*p* = 0.399)

### Knowledge and experience performing cardiopulmonary resuscitation

A one-way ANOVA revealed that knowledge scores differed significantly by doctor’s experience performing pCPR (*F* = 4.37, *p* = 0.006). Post-hoc comparisons revealed that doctors who had never performed pCPR had significantly lower knowledge compared to doctors who had performed pCPR more than 10 times (*p* = 0.006). Knowledge scores increased as the level of experience performing pCPR increased.

### Confidence and anxiety levels

Just over half of doctors (50.3%) felt anxious about the thought of initiating pCPR. Eighteen per cent noted that this anxiety was related to ill-prepared facilities with either a lack of equipment or a lack of trained staff. Almost 10% of doctors felt that pCPR occurred too infrequently, whilst 6% of them felt that inadequate training levels contributed to their high anxiety levels.

Thirty five per cent of doctors did not feel confident in their ability to perform pCPR.

### Confidence levels in performing paediatric cardiopulmonary resuscitation and grades of doctor

A chi-square test was used to investigate the association between confidence in performing pCPR and grade of the doctor. The results showed no significant association between confidence and experience level of doctor (*χ*^2^ = 11.15, *p* = 0.516, Cramer’s *V* = 0.185). Although the results showed no significant association, when looking at the proportions, consultants were the most confident followed by registrars, whereas MO grades 2 and 3 and interns were the least confident.

### Association of confidence levels and courses

Chi-square tests revealed that confidence was not associated with having done BLS, APLS, PHPLS or DIPEC courses. However, doctors who completed the PALS course were significantly more likely to report being confident in performing pCPR than those who did not complete this course (*p* = 0.011). In addition, doctors who completed the ATLS course were significantly more likely to report being confident in performing pCPR than those who did not complete this course (*p* = 0.019).

### Knowledge of equipment availability

More than 85% of doctors were certain that the following equipment items were available: oxygen cylinders; electric/wall suction apparatus; suction catheter; adrenalin injection 1 mg/mL; dextrose 50% intravenous solution; sodium chloride 0.9% 1 L; defibrillator and an emergency trolley with a lockable medicine drawer and accessories.

Doctors were most uncertain (> 35%) about the availability of paediatric nasopharyngeal airways, paediatric oropharyngeal airways, umbilical vein catheters, thermometer, low reading, curved stylet and infant laryngoscope with spare bulbs and batteries. The most common equipment reported as unavailable (around one-third of doctors thought they were unavailable) were umbilical vein catheters, intraosseous needles, heat source and paediatric resuscitation algorithms clearly visible (see Table 5).

**TABLE 5:** Availability of equipment required during paediatric cardiopulmonary resuscitation from the primary health care doctors’ perspective.

Equipment	Yes (%)	No (%)	Uncertain (%)
Paediatric endotracheal tubes of various sizes	68.5	6.2	25.3
Curved stylet	51.6	13.0	35.4
Infant laryngoscope with spare bulbs and batteries	58.0	6.8	35.2
Infant and paediatric bag-valve-masks	83.2	2.5	14.3
Oxygen cylinder	98.8	0.0	1.2
Infant facemasks	80.2	2.5	17.3
Paediatric nasopharyngeal airways	30.4	11.2	58.4
Paediatric oropharyngeal airways	58.0	2.5	39.5
Umbilical vein catheters	24.1	29.6	46.3
Intraosseous needles	32.9	31.1	36.0
Heat source	44.1	32.9	23.0
Thermometer, low reading	42.0	16.7	41.4
Electric/wall suction apparatus	87.7	4.3	8.0
Suction catheter	90.07	0.6	8.6
Adrenalin injection 1 mg/ml	90.7	2.5	6.8
Dextrose 50% intravenous solution	98.1	0.6	1.2
Sodium chloride 0.9% 1 L	98.8	0.6	0.6
Defibrillator	95.1	1.9	3.1
Emergency trolley with a lockable medicine drawer and accessories	96.3	1.2	2.5
Paediatric resuscitation algorithms clearly visible	50.0	27.2	22.8
Gloves: small, medium, large – at least one pair of same size	81.5	12.3	6.2

## Discussion

The study findings indicate that doctors working in the PHC sector of the Cape Metropole do not meet the required resuscitation council of SA standards in terms of knowledge. Not only is the knowledge waning, but pCPR appears to be performed infrequently within the facilities. Confidence levels in performing pCPR are also low, and this is associated with low knowledge levels on pCPR, infrequent clinical presentations of patients requiring pCPR, as well as ill-prepared healthcare facilities. Furthermore, doctors have poor knowledge of the availability of equipment required to perform pCPR within their facilities.

A low knowledge score of pCPR has been found in this study, with an average knowledge score of 61.7%, which is significantly lower than the general pass mark of 80.0% for most CPR courses. Research evidence shows that a lack of knowledge of the guidelines results in an inability to apply pCPR guidelines correctly, thereby resulting in poorer patient outcomes.^1,2,3^

It is well documented in studies performed globally that knowledge wanes with time.^8,19^ This is reflected in this study with knowledge levels decreasing as the grade of doctor (which is a proxy for length of time since last exposure to formal training) increased with grade 3 MOs obtaining the lowest grading on the questionnaire. However, consultants and registrars performed better on the questionnaire than MO grades 2 and 3. This finding can possibly be attributed to their involvement in post-graduate training as well as the finding that more consultants and registrars had done APLS and PALS courses compared to any other grades of doctor. This suggests that knowledge decreases with time since graduation, but education/training may increase and sustain it. Considering that 28.4% of the participants were interns and community service doctors, and that these are not permanent staff, knowledge levels may be lower in PHC settings run only by MOs. This may be the case in many PHC facilities across the WC and South Africa. A possible solution to this problem would be regular training within facilities, with a focus on junior and non-permanent staff members. Furthermore, incentives to regular course attendance could be offered, such as unpaid time off work to attend pCPR courses as well as monetary subsidisation on courses.

International literature shows that a large proportion of doctors only manage cardiac arrest on an average twice a year,^20^ with South African studies showing that doctors who had more exposure to CPR cases performed better in CPR questionnaires compared to those who did not.^21^ The latter concurs with the findings in this study that suggest that doctors do not see many patients requiring pCPR regularly. This, coupled with the findings of low knowledge scores amongst doctors who performed pCPR less regularly or not at all, fits in with studies showing that less experience is associated with lower knowledge scores.^22^ Within the context of this study, this will likely have a negative impact on patient outcomes. pCPR training in the form of regular simulated training and drills, course attendance and CPR algorithms in clear view is advised to enhance recollection of pCPR.

International studies show that the provision of more training in the form of pCPR courses results in a significant increase in resuscitated patients surviving to discharge and having improved neurological outcomes.^1,2^ Whilst this study does not show any association that attending a pCPR course improves knowledge of pCPR, there is a trend related to attendance of the BLS course and higher knowledge scores. The study also shows that the overall knowledge of pCPR amongst doctors is low, regardless of whether they ever attended a course or not. A possible explanation could be that doctors do not perform pCPR frequently, thereby resulting in poor knowledge retention.

The doctors in this study were specifically lacking in knowledge related to rates of rescue breathing and heart rate needed to initiate pCPR, management of VF, as well as knowledge regarding chest compressions. The lack of knowledge on VF is particularly concerning as it is a potentially reversible cause of cardiac arrest. Furthermore, knowledge of rescue breathing and heart rate needed to perform pCPR as well as knowledge regarding chest compressions and the management of VF are integral core aspects of pCPR and performing it incorrectly will lead to poorer patient outcomes.^23^ The study results, therefore, show that doctors lack knowledge on important aspects of pCPR. This is likely to be the result of having never attended a pCPR training course, having last attended a course more than two years prior to the study or performing pCPR infrequently in practice to sustain their skills.

Confidence levels amongst doctors in performing pCPR were low, and those who were confident had higher knowledge levels, even though the overall average knowledge score amongst doctors was low. This suggests that some doctors may be overconfident in their ability to perform pCPR. The results also show that higher confidence levels are associated with attending a paediatric life support course, concurring with findings in the literature.^11^ This emphasises the importance of pCPR course attendance.

Just over 50% of doctors felt anxious about performing pCPR with the main reasons for anxiety noted to be ill-prepared healthcare facilities, inadequate training and the fact that the need to perform pCPR was too infrequent. The latter fits in with the finding of low levels of frequency in performing pCPR.

There is no correlation in studies performed abroad between the general medical experience of the healthcare professional and CPR knowledge and performance.^7^ No studies were found comparing this in South Africa. There is no significant association between confidence and grade of the doctor in this study; however, the findings showed that consultants were the most confident followed by registrars, whereas MO grades 2 and 3 and interns were the least confident. This may be explained by the fact that MO grades 2 and 3 have the lowest knowledge levels compared to the registrars and consultants because they have lower education levels and had lower course attendance levels, whilst the interns, being the most junior, had the lowest experience levels. However, confidence is a self-reported perception and not objectively measurable, particularly because this study did not show statistically significant differences between groups.

Regarding the knowledge of equipment availability, the study shows that participants were most sure of the items in their facility being present that were general items used regularly as well as in adult CPR. Participants were most uncertain about the availability of items specifically needed for pCPR, suggesting they have never used or very seldom use these items at their facility – a finding that may be explained by the infrequent need for pCPR at facilities studied and also that doctors are not in charge of asset control within these facilities. However, considering the low knowledge levels amongst doctors, this is a concerning finding.

## Conclusion

In conclusion, the study shows that PHC doctors are poorly prepared to perform pCPR in their facilities. Regardless of their category, the doctors exhibited low levels of knowledge on how to perform pCPR, lacked confidence levels in their abilities to do so, and poor knowledge of the equipment available in their facilities to adequately perform pCPR. Considering that these findings have implications for poorer patient outcomes amongst paediatric patients presenting with cardiorespiratory arrest, it is recommended that doctors in PHCFs attend emergency care courses regularly in order to maintain their pCPR skills. Recertification in these courses every two years should be mandatory amongst all healthcare workers within PHCFs. Additional measures should be put in place within facilities to enhance recollection of knowledge during pCPR, such as clearly visible pCPR algorithms in the resuscitation room of the facility. Furthermore, doctors should be regularly educated on the locations and availability of equipment required for pCPR within their facility.

Further research is needed to determine the outcomes of cases of pCPR performed within PHCFs. This could be done utilising data from recurring morbidity and mortality meetings. Regular audits of the equipment available for pCPR within healthcare facilities are also advised.

### Limitations

The questionnaire was not a standardised test of doctors’ pCPR knowledge as it was not developed or measured against an accepted gold standard. Knowledge was tested on paper as self-reported competence and not by observing pCPR performance is a further limitation of the study. There was insufficient data with regard to variables measured when comparing knowledge per grade of a doctor, given that we do not know how long the MOs have been in their current position or when the consultants graduated. The same limitation applies to registrars. Furthermore, only 52% of registrars partook in the study that can cause bias or skew the results. Registrars who opted into the study may have had a special interest in pCPR or felt more confident because they had attended a pCPR course, thereby introducing a self-selection bias. This was not the case with the other categories of doctors as all but two of them consented to partake in the study. Limitation to assessing confidence levels is that it is subjective as there is no formal tool currently developed.
